# Geochemical data from Angamuco, Michoacán, Mexico

**DOI:** 10.1016/j.dib.2018.12.071

**Published:** 2018-12-30

**Authors:** Anna S. Cohen, Daniel E. Pierce

**Affiliations:** a Department of Sociology, Social Work, and Anthropology, Utah State University, USA; b Archaeometry Laboratory, University of Missouri, USA

## Abstract

Included here are geochemical concentrations (ppm) of ceramic artifacts and clay samples from the archaeological site of Angamuco, Mexico. Additional data include maps and photographs of the ceramic samples. Concentrations were measured via Instrumental Neutron Activation Analysis and are available here as Appendix B. These data complement the discussions and interpretations in “Geochemical Analysis and Spatial Trends of Ceramics and Clay from Angamuco, Michoacán” [1].

**Specifications table**

**Table:** 

Subject area	*Archaeology*
More specific subject area	*Geochemistry*
Type of data	*Maps, photographs, compositional plots, tables*
How data was acquired	*Instrumental Neutron Activation Analysis, GAUSS 8.0*
Data format	*Raw and analyzed*
Experimental factors	*Sherds were cut, cleaned of any adhering soil and paints, and dried, before being crushed into a fine powder. Clays were fired into briquettes at 700* °*C before pulverization.*
Experimental features	*Compositional analysis of ceramic paste*
Data source location	*Angamuco, Michoacán, Mexico* (237634.01, 2166109.92)
Data accessibility	*Data are with this article in*Appendix B.
Related research article	*Cohen, A.S., Pierce, D.E., Fisher, C.T. (2019) Geochemical Analysis and Spatial Trends of Ceramics and Clay from Angamuco, Michoacan. Journal of Archaeological Science: Reports 23:216-230.*https://doi.org/10.1016/j.jasrep.2018.10.025

**Value of the data**

• Elemental concentrations of chemical elements via INAA can provide insight into ancient ceramic production activities.
• Comparison of INAA data can be used to evaluate hypotheses about ancient trade and exchange.
• Statistical analyses of elemental concentrations may be compared to INAA and petrographic data in the region.

## 1 Data

Included in this dataset is additional information about the broader Angamuco ceramic sample (Table 1, Table 2; see [1] Figs. 1,2 for regional maps). Also included are photographs documenting clay sample collection, the processes of raw clay preparation (Fig. 1, Fig. 2), and examples of the ceramic samples (Appendix A). The results of the Instrumental Neutron Activation Analysis (INAA) performed at the University of Missouri Research Reactor (MURR) are subsequently presented in the form of principal component analyses characterizing the sample in aggregate (Table 3), discriminant analyses in which compositional groups are differentiated (Fig. 3). Final compositional group assignment can be found in Appendix B along with the results of bootstrapped Mahalanobis distance calculations demonstrating the likelihood of compositional group membership. We have also compared raw clay compositions from the Angamuco region to archived data from the nearby Lake Páztcuaro vicinity (Figs. 1 and 4 in [1]; Fig. 4, Table 4). These comparisons show the relationships between compositional group and clays in the region.

**Table 1:** Total ceramic artifacts recovered at Angamuco.

**Spatial Context**	**No. of sherds**
Area A	16,050
Area B	12,125
Area C	21,159
Area D	4934
Area E	5838
Area F	5285
Area G	768
Pedestrian Survey	6111
**Total**	**72,270**

**Table 2:** Archaeological sample contexts.

**Location**	**Time Period**	**Context**	**No. of samples**
Area A	Late Postclassic	Elite domestic	64
	Classic	Partial burial	4
Area B	Late Postclassic	Large Building	31
Area C	Late Postclassic	Ceremonial	95
Area D	Late Postclassic	Ceremonial	18
Area E	Early Postclassic	Domestic	32
Area F	Middle Postclassic	Domestic	52
Area G	Middle Postclassic	Domestic	4
		**Total**	**300**

**Fig. 1:**
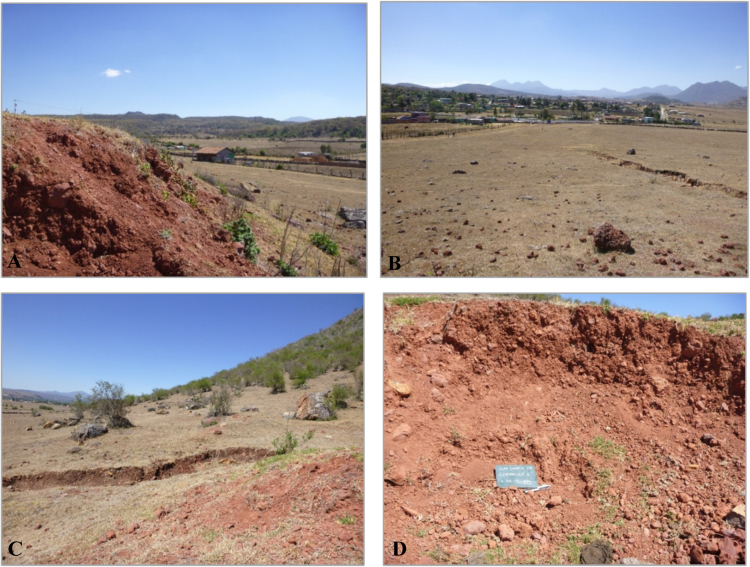
Clay sample 16 (LPB 301), Corrales: A-north view; B-east view; C-west view; D-south view (for map location, see Fig. 2 in [1]).

**Fig. 2:**
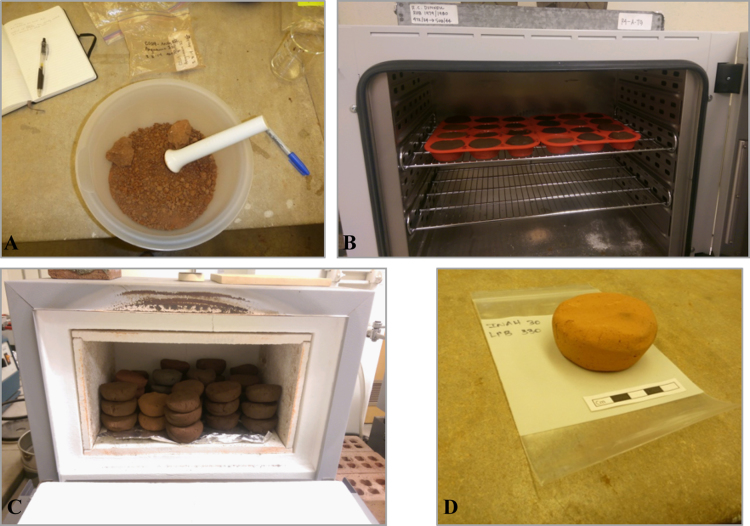
Briquette production: A. Breaking clay with a pestle; B. Briquettes in silicon molds in the drying oven; C. Briquettes in the furnace; D. Fired briquette.

**Table 3:** Elemental Loadings for the pottery sample on Principal Component Axes 1 through 7.

Variable	Mean	PC1	PC2	PC3	PC4	PC5	PC6	PC7
Na	9080.53	−0.112	0.035	0.312	−0.079	−0.309	−0.416	0.092
Al	99,937.10	0.019	−0.083	−0.012	0.070	0.148	−0.088	−0.056
K	8159.28	0.071	0.245	0.293	−0.229	−0.060	−0.154	0.461
Ca	15,976.41	−0.293	−0.083	0.502	0.064	0.017	−0.277	−0.147
Sc	16.19	0.016	−0.181	0.007	0.188	0.139	−0.152	−0.004
Ti	5761.95	0.054	−0.171	0.000	0.132	0.239	−0.209	0.021
V	123.56	0.054	−0.197	−0.027	0.038	0.185	−0.137	0.061
Cr	131.20	0.056	−0.210	−0.120	0.107	0.193	−0.111	0.641
Mn	750.13	0.262	−0.479	0.216	−0.342	−0.275	0.190	−0.014
Fe	49,883.95	0.083	−0.213	0.032	0.102	0.216	−0.203	−0.012
Co	19.63	0.219	−0.480	0.154	−0.201	−0.091	0.071	0.036
Zn	79.54	0.136	−0.105	0.133	0.096	0.191	−0.188	−0.096
Rb	49.06	0.141	0.273	0.305	−0.251	0.150	0.195	0.069
Sr	301.69	−0.294	0.001	0.471	0.081	0.226	0.162	−0.038
Zr	155.30	0.178	0.021	0.019	0.013	0.166	−0.079	0.055
Sb	0.19	0.279	0.015	0.013	−0.102	0.261	−0.129	−0.162
Cs	1.79	0.272	0.105	−0.036	−0.291	0.188	−0.086	−0.390
Ba	652.33	−0.051	−0.012	0.229	0.100	0.418	0.558	0.099
La	22.98	0.112	−0.051	0.071	0.227	−0.045	0.054	0.020
Ce	52.91	0.181	−0.120	0.070	0.009	−0.141	0.201	0.032
Nd	22.60	0.110	−0.040	0.074	0.275	−0.087	0.086	−0.018
Sm	5.28	0.126	−0.009	0.072	0.244	−0.102	0.034	−0.021
Eu	1.19	−0.007	−0.164	0.006	0.262	−0.047	0.050	−0.023
Tb	0.73	0.152	0.060	0.129	0.262	−0.192	0.041	−0.097
Dy	4.34	0.148	0.066	0.104	0.288	−0.142	0.004	−0.065
Yb	2.44	0.170	0.077	0.105	0.211	−0.150	0.048	−0.073
Lu	0.34	0.172	0.070	0.088	0.228	−0.135	0.047	−0.063
Hf	6.58	0.195	0.063	0.052	0.011	0.109	−0.104	0.006
Ta	1.22	0.276	0.169	0.137	0.021	0.082	−0.147	−0.084
Th	5.74	0.255	0.163	0.047	0.016	0.132	−0.062	−0.002
U	1.46	0.311	0.234	0.010	0.105	−0.093	0.029	0.314
Eigenvalues:		0.361	0.129	0.091	0.034	0.028	0.022	0.015
Total Variation explained:		47.79%	17.09%	12.04%	4.52%	3.70%	2.92%	1.92%

**Fig. 3:**
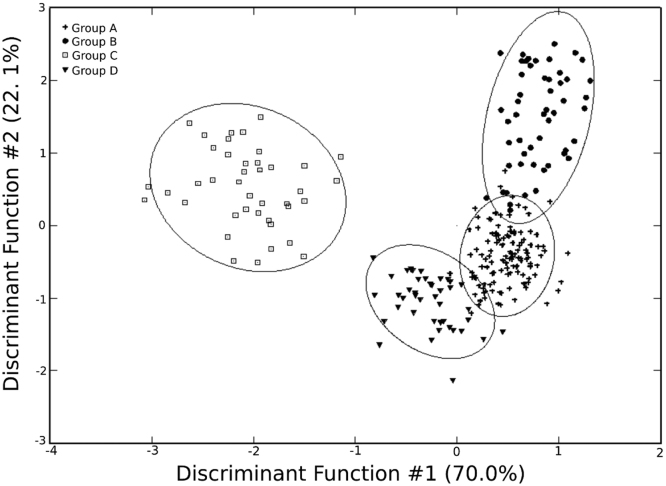
Results of canonical discriminant analysis (including raw clay samples).

## 2 Experimental design, materials and methods

### 2.1 Location

These data consist of compositional analysis from 300 archaeological ceramics and 30 raw clay specimens from the site of Angamuco in the state of Michoacán, Mexico (Figs. 1,2 in [1]). Located approximately 9 km southeast from the Purépecha (Tarascan) imperial capital of Tzintzuntzan within the Lake Pátzcuaro Basin, Angamuco was occupied from at least the Classic through the Late Postclassic periods (c. 250–1530 CE) [2], [3]. This site is presumed to have already been a large civic center prior to Purépecha development and may have played a role in regional development and interaction. Angamuco covers an area of greater than 26 km² and includes over 40,000 architectural features [4], [5]. Recently, the area has been the subject of the large scale survey and excavation project, “Legacies of Resilience: The Lake Pátzcuaro Basin Archaeological Project” [3], [6], [7], [8], [9], [10], [11], [12], and has produced a wide array of ceramic, lithic, and other artifacts [2] (Table 1). Aside from the 300 archaeological specimens sampled for geochemical analysis, 30 raw clay deposits were sampled from the immediate vicinity (Fig. 2 in [1]).

### 2.2 Sample description

The overall purpose of data collection was to test the common presumption that polity development involves the co-opting of existing local institutions and subsequently creating new administrative, economic, and ideological systems [13], [14]. More specifically, we have used ceramic provenance analyses to assess the incorporation of the Angamuco region during Purépecha Empire development through the identification of diachronic and synchronic pottery consumption patterns. Archaeological samples were chosen for geochemical analysis via non-randomly stratified sampling to sufficiently represent the typological, spatial, and temporal variability at Angamuco. In total, 300 ceramic sherds were chosen from seven different areas of the site (Table 2 and Fig. 2 in [1]) including public (e.g. plazas) and private (e.g. rooms within domestic contexts) spaces. Samples from both pre-Purépecha (Classic to Middle Postclassic periods, c. 250–1350 CE) and Purépecha (c. 1350–1530 CE) era contexts to assess temporal variability (see [2, pp. 159–164] for discussion on contextual dating). Finally, thirty raw clay deposits were selected based upon their likelihood of availability to prehistoric potters. Samples were chosen from areas in close proximity to Angamuco, as 50% of prehistoric and ethnographic potters collect clays within 2 km of workshops [15], [16], [17] (Fig. 2 in [1]). Samples were typically collected from exposed profiles and GPS coordinates were recorded (Fig. 1).

### 2.3 INAA raw clay and archaeological sample preparation

Using standard protocol for INAA sample preparation [18], [19], [20], [21] all clays (n=30) and archaeological samples (n=300) were prepared at the Archaeometry Laboratory at the University of Missouri Research Reactor. Clays were fired as briquettes at 700 °C and then ground into a fine powder using an agate mortar and pestle using procedures described by [22] (Fig. 2). Samples of 1 cm^2^ were removed using a silicon carbide burr from archaeological specimen for analysis. In doing so, all adhering soil, glaze, slip, and/or paints were removed, to minimize the error produced by the inadvertent measurement of non-paste compositions. Specimens were then washed in deionized water and dried before being ground into a fine powder. A sample of 150 mg of powder from each specimen was then sealed into a high-density polyethylene vile, while a second sample of 200 mg was measured into a high-purity quartz vial for long irradiation. Standards in the form of Basalt Rock and Coal Fly Ash from the National Institute of Standards and Technology (NIST) as well as control samples of Obsidian Rock and Ohio Red Clay were also utilized.

Irradiation consisted of three separate gamma counts. Following an initial neutron flux of 8 × 10^13^ n cm^−2^ s^−1^ of five seconds was accessed through a pneumatic tube system [18], a gamma count of 720 seconds measured concentrations of nine short-lived elements: aluminum (Al), barium (Ba), calcium (Ca), dysprosium (Dy), potassium (K), manganese (Mn), sodium (Na), titanium (Ti), and vanadium (V). The second larger sample was subjected to a 24-h irradiation at a neutron flux of 5 × 10^13^ n cm^−2^ s^−1^. The sample then decayed for seven days before recording gamma counts of 1800 s using a high-resolution germanium detector. The following medium half-life elements were recorded: arsenic (As), lanthanum (La), lutetium (Lu), neodymium (Nd), samarium (Sm), uranium (U), and ytterbium (Yb). After an additional 4 week decay process, a final count of 8500 s yielded measurements of seventeen long lived elements; cerium (Ce), cobalt (Co), chromium (Cr), cesium (Cs), europium (Eu), iron (Fe), hafnium (Hf), nickel (Ni), rubidium (Rb), antimony (Sb), scandium (Sc), strontium (Sr), tantalum (Ta), terbium (Tb), thorium (Th), zinc (Zn), and zirconium (Zr). Due to the frequency at which its proportion falls below detection limits, Nickel (Ni) was removed from analysis. The remaining 32 elements were recorded as parts per million and included in excel spreadsheets for importation into statistical analysis software.

### 2.4 Multivariate statistical analysis of compositional data

Using GAUSS 8.0 software, a variety of multivariate statistical analyses using base-10 logarithms were utilized to characterize the sample in aggregate, differentiate compositional groups, and compare the Angamuco sample with previously collected archived data (Table 3). A comprehensive discussion of these analytical methods, including principal component analyses, discriminant function analyses, and Mahalanobis distance calculations can be found elsewhere (e.g. [18], [19], [23], [24], [25], [26]. In our analysis, we first began with principal component analysis (Fig. 3 in [1]) per the provenance postulate [27], this was followed by visual inspection of bivariate plots and bootstrapped multi-dimensional Mahalanobis Distance calculations to differentiate compositional groups (Appendix B). These groups were further defined through canonical discriminant analysis (Fig. 3). The geochemical data were also compared to archived data at MURR, most significantly from the Lake Pátzcuaro region [28] through elemental biplots, Mahalanobis distance, and through mean Euclidean distance in multivariate compositional space (Table 3 in [1]). ArcMap 10.3 was utilized to visually assess compositional variability across the landscape using an interpolation based upon the composition of raw clay from Angamuco and the archived Lake Pátzcuaro samples (Fig. 4).

**Fig. 4:**
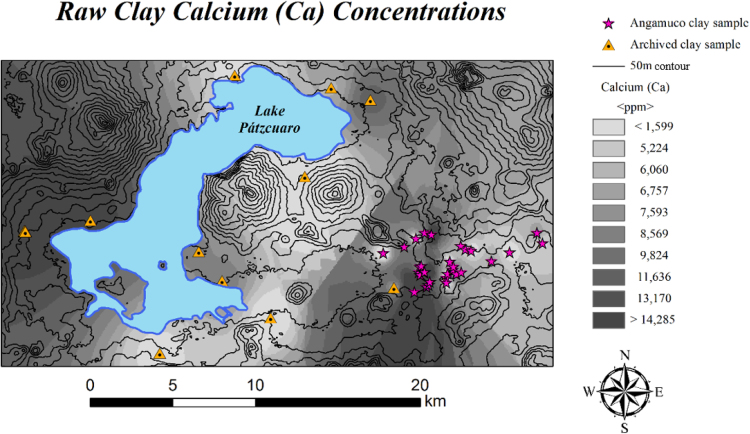
Results of compositional interpolation (calcium) in the Lake Pátzcuaro Basin.

## Figures and Tables

**Fig. 1:**
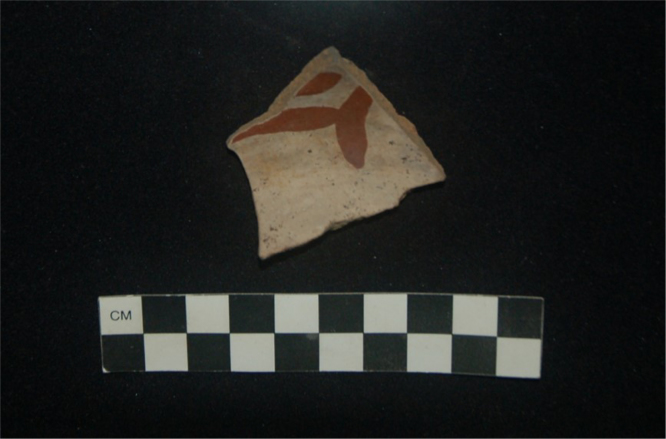
Sample MURR 232. Bichrome bowl fragment from Area A (AN10E25-5).

**Fig. 2:**
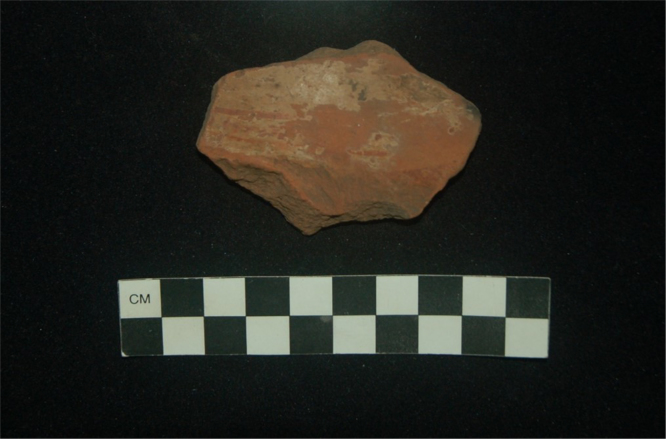
Sample MURR 203. Polychrome tripod bowl fragment from Area B (BN12E10-2c3).

**Fig. 3:**
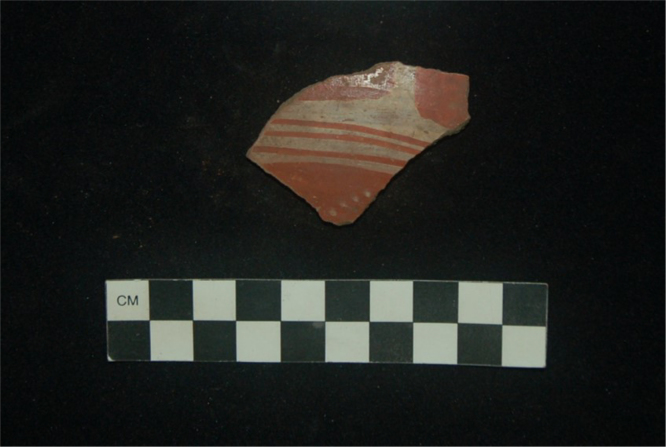
Sample MURR 65. Polychrome jar fragment from Area C (CN12E14-1).

**Fig. 4:**
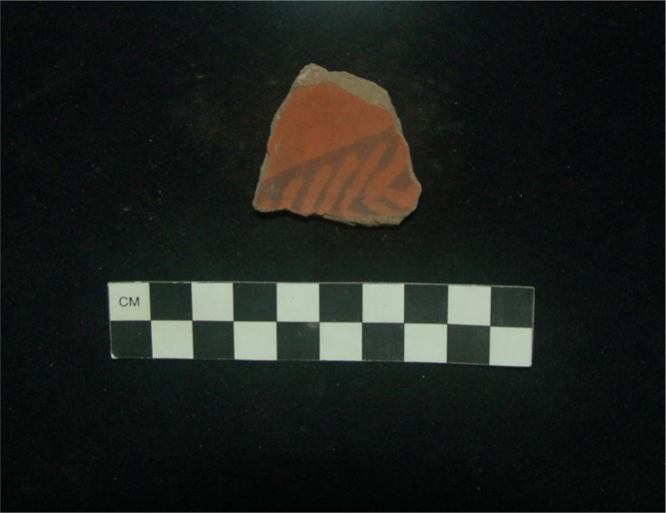
Sample MURR 106. Bichrome negative bowl fragment from Area C (CN12E16-7).

**Fig. 5:**
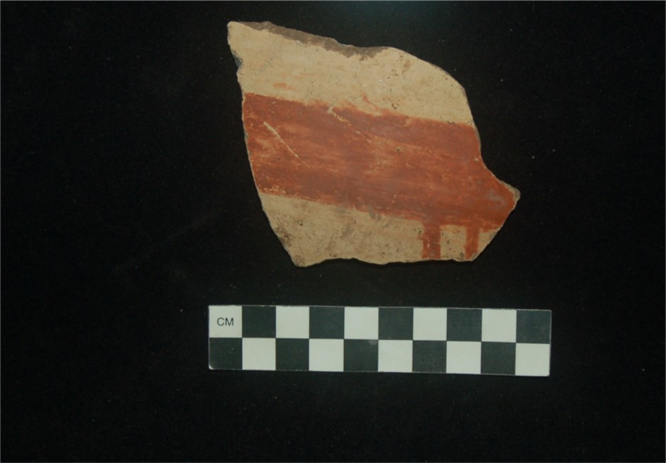
Sample MURR 5. Bichrome fragment from Area D (DN0E2–5).

**Fig. 6:**
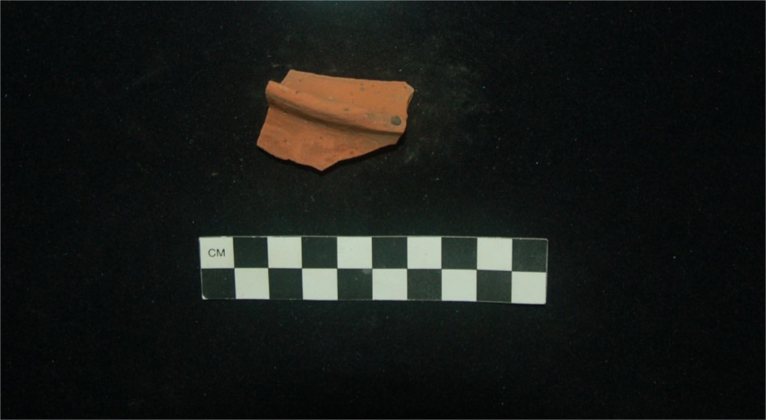
Sample MURR 112. Annual base fragment from Area E (E1N0E0–1).

**Fig. 7:**
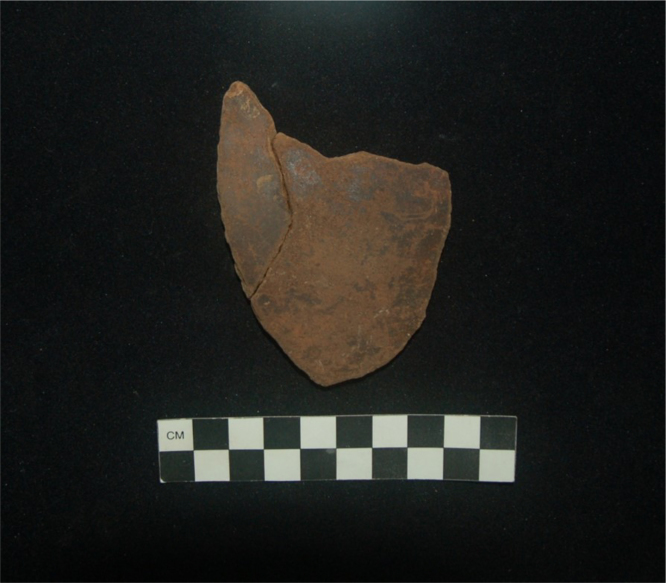
Sample MURR 143. Eroded jar fragment from Area F (F1S1E0–3F1).

**Fig. 8:**
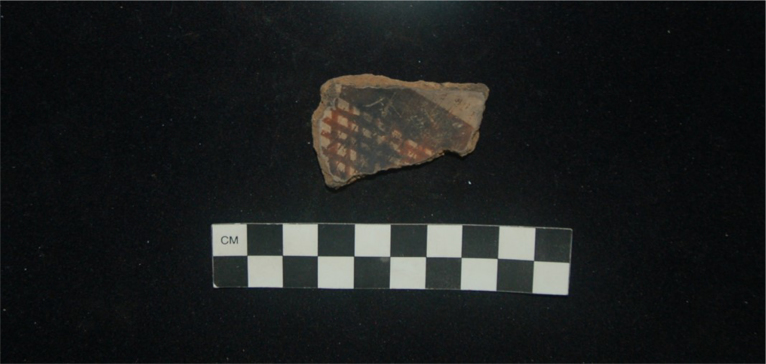
Sample MURR 178. Bichrome fragment with cross-hatching from Area F (F6S3E0–15).

**Table:** 

ANID	Group A	Group B	Group C	Group D	Best Group
LPB001	46.591	3.442	0.000	0.298	Group A
LPB003	88.834	7.550	0.000	0.088	Group A
LPB006	71.352	3.260	0.000	0.023	Group A
LPB009	44.322	16.089	0.000	0.001	Group A
LPB010	37.889	0.862	0.003	7.033	Group A
LPB012	56.933	1.065	0.000	2.435	Group A
LPB017	76.368	14.841	0.000	0.003	Group A
LPB019	92.402	2.690	0.000	0.269	Group A
LPB023	93.740	4.715	0.000	0.027	Group A
LPB025	44.528	1.810	0.000	0.004	Group A
LPB026	11.014	2.587	0.025	0.343	Group A
LPB027	17.120	0.386	0.000	7.583	Group A
LPB028	6.434	0.178	0.000	0.056	Group A
LPB030	46.461	2.308	0.000	0.003	Group A
LPB031	34.902	3.213	0.000	0.071	Group A
LPB035	70.574	15.679	0.000	0.004	Group A
LPB040	0.201	0.073	0.001	13.804	Group D
LPB041	84.754	2.480	0.000	0.130	Group A
LPB042	70.992	6.493	0.000	0.002	Group A
LPB045	55.964	13.232	0.000	0.002	Group A
LPB047	91.358	4.345	0.000	0.040	Group A
LPB048	96.359	10.068	0.000	0.015	Group A
LPB051	39.037	9.847	0.003	0.085	Group A
LPB052	60.380	3.223	0.000	0.227	Group A
LPB053	39.673	4.540	0.000	0.002	Group A
LPB054	10.986	8.127	0.000	0.000	Group A
LPB056	64.773	2.728	0.000	0.006	Group A
LPB058	86.957	4.866	0.000	0.019	Group A
LPB059	76.526	15.573	0.000	0.009	Group A
LPB062	54.249	15.813	0.002	0.019	Group A
LPB065	73.751	3.915	0.000	0.276	Group A
LPB074	76.233	3.211	0.000	0.053	Group A
LPB077	33.511	0.776	0.000	5.167	Group A
LPB079	71.617	3.244	0.002	1.074	Group A
LPB082	49.147	1.156	0.000	0.230	Group A
LPB083	64.615	5.745		0.000	0.096
LPB085	86.417	5.518	0.000	0.150	Group A
LPB089	39.076	17.300	0.001	0.002	Group A
LPB091	42.966	13.952	0.002	0.008	Group A
LPB094	78.287	10.955	0.001	0.049	Group A
LPB096	3.811	0.717	0.000	0.000	Group A
LPB114	3.812	0.124	0.000	0.002	Group A
LPB121	22.449	0.505	0.002	14.240	Group A
LPB128	3.166	5.573	0.023	0.031	Group B
LPB129	36.983	6.882	0.004	0.047	Group A
LPB140	62.356	2.469	0.000	0.182	Group A
LPB141	95.714	3.221	0.000	0.456	Group A
LPB143	11.006	0.820	0.000	0.005	Group A
LPB144	11.385	2.207	0.000	0.051	Group A
LPB149	14.290	35.611	0.000	0.000	Group B
LPB151	81.581	2.855	0.000	0.090	Group A
LPB158	90.284	4.754	0.000	0.042	Group A
LPB159	10.703	6.220	0.001	0.004	Group A
LPB160	81.976	5.741	0.000	0.013	Group A
LPB161	49.694	2.798	0.000	0.002	Group A
LPB162	55.235	2.272	0.000	0.009	Group A
LPB163	43.214	1.073	0.000	0.015	Group A
LPB164	32.538	12.910	0.000	0.001	Group A
LPB166	8.102	0.184	0.000	17.718	Group D
LPB167	25.831	0.386	0.001	15.510	Group A
LPB168	60.803	1.062	0.000	2.197	Group A
LPB170	98.689	4.770	0.000	0.123	Group A
LPB171	51.916	6.037	0.004	0.256	Group A
LPB172	37.449	20.458	0.000	0.000	Group A
LPB173	70.989	2.305	0.000	0.412	Group A
LPB174	92.709	4.194	0.000	0.088	Group A
LPB175	54.512	1.531	0.000	0.264	Group A
LPB177	34.233	2.033	0.000	0.001	Group A
LPB179	73.696	10.661	0.001	0.037	Group A
LPB180	41.448	6.174	0.000	0.001	Group A
LPB181	88.290	2.896	0.000	0.131	Group A
LPB182	27.825	0.473	0.001	12.796	Group A
LPB184	41.191	3.120	0.000	0.067	Group A
LPB185	40.923	1.184	0.002	3.419	Group A
LPB187	47.440	0.801	0.001	6.967	Group A
LPB188	70.714	1.385	0.000	0.683	Group A
LPB189	60.793	13.549	0.001	0.031	Group A
LPB190	5.083	0.162	0.028	23.232	Group D
LPB191	46.997	2.582	0.000	0.019	Group A
LPB193	88.732	5.643	0.001	0.183	Group A
LPB194	95.294	8.113	0.000	0.032	Group A
LPB201	7.846	0.577	0.000	0.738	Group A
LPB202	85.760	6.352	0.000	0.006	Group A
LPB204	84.769	14.777	0.000	0.006	Group A
LPB205	1.143	26.145	0.000	0.000	Group B
LPB206	69.971	9.968	0.000	0.012	Group A
LPB211	95.946	5.506	0.000	0.023	Group A
LPB212	66.836	2.261	0.002	2.055	Group A
LPB213	0.743	15.233	0.000	0.000	Group B
LPB214	57.957	1.869	0.003	2.748	Group A
LPB221	29.710	0.646	0.000	3.680	Group A
LPB223	15.313	42.156	0.001	0.000	Group B
LPB232	78.904	11.361	0.001	0.027	Group A
LPB236	24.738	9.150	0.000	0.002	Group A
LPB239	72.024	5.861	0.000	0.003	Group A
LPB240	92.598	10.974	0.000	0.018	Group A
LPB243	86.601	10.243	0.001	0.057	Group A
LPB244	65.253	2.112	0.000	0.014	Group A
LPB252	89.284	8.372	0.000	0.039	Group A
LPB255	91.016	12.982	0.000	0.013	Group A
LPB256	5.111	18.917	0.000	0.000	Group B
LPB257	16.921	0.592	0.002	9.986	Group A
LPB259	69.757	3.546	0.002	0.499	Group A
LPB260	6.020	0.360	0.000	0.437	Group A
LPB261	26.408	0.915	0.001	5.915	Group A
LPB264	48.803	1.033	0.000	0.052	Group A
LPB265	39.324	2.925	0.000	0.002	Group A
LPB267	81.314	15.945	0.000	0.008	Group A
LPB270	88.693	8.304	0.000	0.005	Group A
LPB272	17.169	0.438	0.000	0.189	Group A
LPB276	22.974	1.249	0.014	3.163	Group A
LPB279	55.978	1.475	0.000	0.228	Group A
LPB281	7.240	4.275	0.003	0.280	Group A
LPB284	13.730	0.879	0.000	0.512	Group A
LPB286	45.637	0.868	0.001	6.636	Group A
LPB287	83.612	8.095	0.000	0.038	Group A
LPB290	30.206	4.709	0.001	0.244	Group A
LPB292	4.583	0.145	0.000	0.006	Group A
LPB294	78.072	2.195	0.001	1.805	Group A
LPB298	30.052	1.128	0.000	1.730	Group A
LPB299	55.683	1.300	0.000	0.041	Group A
LPB300	49.550	8.633	0.000	0.001	Group A

**Table:** 

**ANID**	**Group A**	**Group B**	**Group C**	**Group D**	**Best Group**
LPB002	18.170	31.602	0.001	0.001	Group B
LPB007	0.000	45.456	0.000	0.000	Group B
LPB008	0.021	18.514	0.000	0.000	Group B
LPB020	0.435	59.169	0.000	0.000	Group B
LPB022	0.000	50.364	0.000	0.000	Group B
LPB024	0.000	99.396	0.000	0.000	Group B
LPB032	0.108	80.862	0.000	0.000	Group B
LPB033	0.000	13.646	0.000	0.000	Group B
LPB034	0.000	8.238	0.000	0.000	Group B
LPB038	0.000	49.792	0.000	0.000	Group B
LPB039	0.000	71.752	0.000	0.000	Group B
LPB043	0.008	46.511	0.000	0.000	Group B
LPB049	16.516	28.853	0.000	0.000	Group B
LPB057	0.000	61.900	0.000	0.000	Group B
LPB063	0.000	97.515	0.000	0.000	Group B
LPB068	3.331	10.074	0.010	0.005	Group B
LPB070	0.017	65.665	0.000	0.000	Group B
LPB071	2.650	15.628	0.002	0.005	Group B
LPB072	0.000	38.861	0.000	0.000	Group B
LPB073	0.000	59.474	0.000	0.000	Group B
LPB076	0.000	64.315	0.000	0.000	Group B
LPB086	0.582	52.308	0.000	0.000	Group B
LPB087	0.027	94.185	0.000	0.000	Group B
LPB088	15.205	25.172	0.001	0.000	Group B
LPB097	1.621	28.783	0.000	0.000	Group B
LPB098	0.000	48.086	0.000	0.000	Group B
LPB099	0.000	40.939	0.000	0.000	Group B
LPB101	8.373	28.178	0.000	0.000	Group B
LPB103	0.000	28.953	0.000	0.000	Group B
LPB104	0.000	0.760	0.000	0.000	Group B
LPB107	0.000	6.884	0.000	0.000	Group B
LPB134	0.000	31.863	0.000	0.000	Group B
LPB135	0.000	70.543	0.000	0.000	Group B
LPB136	0.000	85.518	0.000	0.000	Group B
LPB137	0.000	26.309	0.000	0.000	Group B
LPB138	0.031	15.834	0.001	0.000	Group B
LPB153	0.000	66.634	0.000	0.000	Group B
LPB156	0.000	30.411	0.000	0.000	Group B
LPB176	0.009	38.777	0.000	0.000	Group B
LPB197	0.000	93.746	0.000	0.000	Group B
LPB208	0.001	27.543	0.000	0.000	Group B
LPB210	0.000	13.548	0.000	0.000	Group B
LPB215	1.319	58.238	0.000	0.000	Group B
LPB216	0.000	68.330	0.000	0.000	Group B
LPB218	0.000	20.292	0.000	0.000	Group B
LPB224	0.000	75.787	0.000	0.000	Group B
LPB235	0.000	55.759	0.000	0.000	Group B
LPB247	0.000	72.635	0.000	0.000	Group B
LPB251	0.000	79.230	0.000	0.000	Group B
LPB258	0.000	38.891	0.000	0.000	Group B
LPB266	0.000	68.295	0.000	0.000	Group B
LPB291	0.017	70.015	0.001	0.000	Group B

**Table:** 

**ANID**	**Group A**	**Group B**	**Group C**	**Group D**	**Best Group**
LPB013	0.000	0.000	77.397	0.002	Group C
LPB044	0.000	0.000	82.918	0.000	Group C
LPB075	0.000	0.000	30.333	0.000	Group C
LPB090	0.000	0.000	1.686	0.001	Group C
LPB110	0.000	0.000	97.186	0.000	Group C
LPB111	0.000	0.000	9.155	0.000	Group C
LPB112	0.000	0.000	4.885	0.000	Group C
LPB113	0.000	0.000	85.017	0.000	Group C
LPB115	0.000	0.000	68.970	0.000	Group C
LPB116	0.000	0.000	5.191	0.000	Group C
LPB117	0.000	0.000	32.690	0.004	Group C
LPB118	0.000	0.000	39.242	0.000	Group C
LPB119	0.000	0.000	24.533	0.000	Group C
LPB120	0.000	0.000	54.312	0.000	Group C
LPB122	0.000	0.000	49.611	0.000	Group C
LPB123	0.000	0.000	91.054	0.000	Group C
LPB125	0.000	0.000	84.045	0.000	Group C
LPB127	0.000	0.000	84.627	0.001	Group C
LPB130	0.000	0.000	81.755	0.001	Group C
LPB131	0.000	0.000	44.567	0.014	Group C
LPB132	0.000	0.000	54.750	0.000	Group C
LPB152	0.000	0.000	44.010	0.000	Group C
LPB183	0.000	0.000	25.000	0.000	Group C
LPB195	0.000	0.000	55.200	0.000	Group C
LPB198	0.000	0.000	87.576	0.000	Group C
LPB203	0.000	0.000	26.998	0.000	Group C
LPB209	0.000	0.000	70.560	0.000	Group C
LPB219	0.000	0.000	85.285	0.000	Group C
LPB222	0.000	0.000	23.996	0.000	Group C
LPB228	0.000	0.000	22.718	0.089	Group C
LPB234	0.000	0.000	85.776	0.000	Group C
LPB245	0.000	0.000	48.499	0.000	Group C
LPB248	0.000	0.000	10.648	0.000	Group C
LPB250	0.000	0.000	89.261	0.000	Group C
LPB254	0.000	0.000	25.760	0.000	Group C
LPB274	0.000	0.000	12.323	0.000	Group C
LPB278	0.000	0.000	39.491	0.000	Group C
LPB280	0.000	0.000	35.826	0.000	Group C
LPB285	0.000	0.000	26.360	0.000	Group C
LPB293	0.000	0.000	42.165	0.000	Group C
LPB295	0.000	0.000	55.251	0.000	Group C

**Table:** 

**ANID**	**Group A**	**Group B**	**Group C**	**Group D**	**Best Group**
LPB011	2.295	0.043	0.004	58.811	Group D
LPB016	0.011	0.001	0.144	90.044	Group D
LPB018	0.000	0.000	0.128	38.084	Group D
LPB021	0.000	0.000	0.206	49.849	Group D
LPB036	0.000	0.000	0.255	71.008	Group D
LPB037	0.270	0.006	0.063	95.461	Group D
LPB064	0.011	0.001	0.334	84.954	Group D
LPB067	0.373	0.066	0.016	19.668	Group D
LPB069	0.001	0.000	0.033	62.449	Group D
LPB078	0.016	0.003	0.047	70.271	Group D
LPB092	0.665	0.022	0.002	43.146	Group D
LPB095	0.035	0.001	0.135	97.217	Group D
LPB102	0.000	0.000	4.129	6.501	Group D
LPB126	0.212	0.004	0.007	77.792	Group D
LPB133	0.018	0.001	0.072	76.404	Group D
LPB145	0.000	0.000	0.017	31.859	Group D
LPB146	0.000	0.000	1.087	54.646	Group D
LPB147	0.000	0.000	1.122	28.372	Group D
LPB148	0.657	0.019	0.088	63.701	Group D
LPB150	0.000	0.000	0.165	69.794	Group D
LPB154	0.000	0.000	0.160	38.497	Group D
LPB155	0.000	0.000	0.001	0.211	Group D
LPB157	0.065	0.003	0.227	80.891	Group D
LPB165	5.077	0.071	0.001	37.784	Group D
LPB169	0.060	0.012	0.000	8.488	Group D
LPB186	0.015	0.001	0.539	61.524	Group D
LPB192	0.000	0.000	0.240	18.356	Group D
LPB207	0.666	0.012	0.025	87.665	Group D
LPB226	0.038	0.004	0.001	24.163	Group D
LPB227	1.441	0.054	0.024	48.294	Group D
LPB229	0.000	0.000	0.044	29.146	Group D
LPB230	0.003	0.000	0.112	83.915	Group D
LPB233	0.006	0.001	0.000	1.558	Group D
LPB237	0.000	0.000	0.025	1.665	Group D
LPB241	0.000	0.000	0.173	59.675	Group D
LPB246	0.346	0.017	0.057	65.550	Group D
LPB249	0.837	0.016	0.002	54.017	Group D
LPB253	0.978	0.053	0.000	11.249	Group D
LPB263	0.031	0.002	0.528	41.339	Group D
LPB273	0.001	0.000	0.024	58.275	Group D
LPB277	1.014	0.031	0.059	56.786	Group D
LPB296	0.008	0.000	0.219	96.854	Group D
LPB297	0.014	0.001	0.074	88.387	Group D

## Appendix A

**Sample photos of Angamuco sherd fragments submitted for INAA**.

See Fig. 1, Fig. 2, Fig. 3, Fig. 4, Fig. 5, Fig. 6, Fig. 7, Fig. 8 here.

## Appendix B

**Mahalonobis results confirming group membership**.

Membership probabilities(%) for samples in group: Group A.

Probabilities calculated after removing each sample from group.

Membership probabilities (%) for samples in group: Group B.

Probabilities calculated after removing each sample from group.

Membership probabilities (%) for samples in group: Group C.

Probabilities calculated after removing each sample from group.

Membership probabilities (%) for samples in group: Group D.

Probabilities calculated after removing each sample from group.
